# False Positive HIV Diagnoses in Resource Limited Settings: Operational Lessons Learned for HIV Programmes

**DOI:** 10.1371/journal.pone.0059906

**Published:** 2013-03-20

**Authors:** Leslie Shanks, Derryck Klarkowski, Daniel P. O'Brien

**Affiliations:** 1 Médecins Sans Frontières, Amsterdam, The Netherlands; 2 Department of Medicine and Infectious Diseases, Royal Melbourne Hospital, University of Melbourne, Melbourne, Australia; Helmholtz Zentrum Muenchen – German Research Center for Environmental Health, Germany

## Abstract

**Background:**

Access to HIV diagnosis is life-saving; however the use of rapid diagnostic tests in combination is vulnerable to wrongly diagnosing HIV infection when both screening tests give a false positive result. Misclassification of HIV patients can also occur due to poor quality control, administrative errors and lack of supervision and training of staff. Médecins Sans Frontières discovered in 2004 that HIV negative individuals were enrolled in some HIV programmes. This paper describes the result of an audit of three sites to review testing practices, implement improved testing algorithms and offer re-testing to clients enrolled in the HIV clinic.

**Findings:**

In the Democratic Republic of Congo (DRC), Burundi and Ethiopia patients were identified for HIV retesting. In total, 44 false-positive patients were identified in HIV programmes in DRC, two in Burundi and seven in Ethiopia. Some of those identified had been abandoned by partners or started on anti-retroviral therapy or prophylaxis. Despite potential damage to programme reputations, no impact in terms of testing uptake occurred with mean monthly testing volumes stable after introduction of re-testing. In order to prevent the problem, training, supervision and quality control of testing procedures were strengthened. A simple and feasible confirmation test was added to the test algorithm. Prevalence of false positives after introducing the changes varied from zero percent (95% CI 0%–8.2%) to 10.3 percent (95% CI: 7.2%–14.1%) in Burundi and DRC respectively.

**Conclusion:**

False HIV diagnoses were found in a variety of programme settings and had devastating individual consequences. We re-tested individuals in our programmes while instituting improved testing procedures without a negative impact on test uptake. Considering the importance of correct diagnosis to the individual, as well as the resources needed to care for someone with HIV, it is critical to ensure that all patients registered in HIV programmes are accurately diagnosed.

## Introduction

Access to HIV diagnosis is life-saving and essential to confronting the HIV pandemic. The WHO algorithm which uses rapid diagnostic tests (RDT) in combination to diagnose HIV has been critical to scaling up access to life-saving treatment [1]. However the algorithm is vulnerable to wrongly diagnosing HIV infection when both screening tests give a false positive result. This has been documented in a number of countries and is predominantly caused by serological cross reactivity [2]–[6]. Cross reactivity, whereby an antibody will bind to an antigen that differs from its originator antigen, is common and linked to a variety of different phenomena such as immature immune responses, other infections, or autoimmune disorders [7], [8]. Cross reactivity is often signalled by a high rate of discordant results as the cross reacting antibodies are known to react to the test antigens variably [9], [10]. The use of the tie-breaker algorithm, where a third RDT is used to resolve a discordant result, increases the risk of misdiagnosis in these circumstances as it will react unpredictably with the cross-reactive antibodies and therefore the outcome is determined by chance [11]. False positive results are higher in low HIV prevalence contexts as even RDTs with a high specificity can perform poorly. Other reasons for misclassifying individuals with HIV are linked to administrative errors, test storage and transport conditions, poor quality control, and lack of training and supervision of staff.

Médecins Sans Frontières-Operational Centre Amsterdam (MSF) offers free of charge comprehensive HIV care programmes in Sub-Saharan Africa and Asia in both rural and urban settings often in partnership with local Ministries of Health. The problem of misclassification of HIV diagnosis became apparent to MSF staff when falsely diagnosed HIV individuals were identified in programmes in 2004. The experience led us to address quality control procedures, and improve the diagnostic algorithm. This paper uses three country case studies to illustrate how the problem was identified, characterise the profile of individuals falsely identified as HIV positive, describe the programmatic response and illustrate the consequences of the misclassification on individuals affected.

## Methods

An audit of HIV testing in MSF-OCA programmes was done during the period 2004–5 to investigate reasons for false positive HIV tests. This review resulted in the implementation of new policies and procedures which included re-testing high risk individuals to confirm their HIV status. The results of both the audit and the implementation of the new procedures are described here using three country case studies from the Democratic Republic of the Congo (DRC), Burundi and Ethiopia.

The programmes were chosen from amongst 22 programmes in Sub-Saharan Africa that were active during this period. DRC, Burundi and Ethiopia were chosen because they are representative of the overall programmes in MSF during this time period and because data on the individuals re-tested was available.

### Programmatic intervention

#### (i) Quality control

In 2003 and 2004, MSF started to receive reports of HIV negative individuals being discovered in MSF's HIV programmes. One of MSF's earliest HIV programmes in Zambia was the first to signal the problem followed by programmes in Humera, Ethiopia and Bukavu, DRC. MSF's response to these reports was to review testing procedures and quality control. Test kit expiry dates were double checked. Records of transport and storage conditions of test kits were reviewed. Training and supervision of testing centres was intensified to minimise errors in the performance and interpretation of tests or in the handling or labelling of samples. All positive and discordant results at the VCT sites were repeated on venous samples in the laboratory. Quality control for each new box of HIV tests was introduced using known positive and negative controls. The standard procedure of monthly quality control of 20 randomly selected positive and negative samples was reinforced.

#### (ii) Confirmation testing

**Figure 1:**
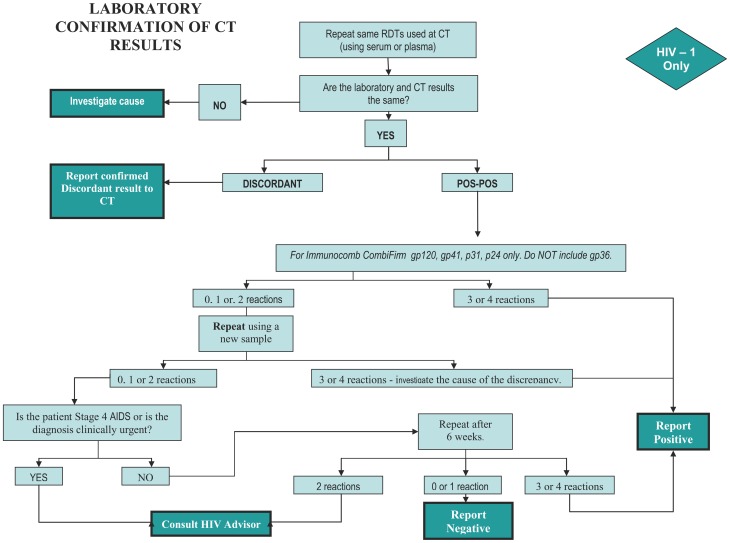
HIV Diagnostic Algorithm using the Orgenics Immunocomb Combfirm® confirmatory test.

In 2005, when it became apparent that false positives were still present despite these measures, the decision was made to introduce confirmation testing. The choice of confirmation test was Western Blot where possible or Orgenics Immunocomb Combfirm® HIV confirmation test (OIC). The use of the latter test is described elsewhere [6]. It has proved feasible for use in peripheral laboratories, takes just two hours to perform and costs five euro. A limitation is that serological confirmation tests are prone to indeterminate results in early seroconversion or severe immunosuppression. A testing algorithm was designed to address these limits (Figure 1).

Clients were told that the screening RDTs indicated the result was positive, but that a confirmatory test was required. Where testing was done from a capillary sample by the counsellor in front of the client, a venous sample was drawn to re-test all discordant and positive results in the laboratory. Most programmes continued to immediately refer those screening positive to the HIV clinic to avoid defaulting. The result of the confirmation test was conveyed to their clinician to be communicated at the next visit. More rarely, where there was a high prevalence of false positives, confirmation testing was done while the client waits. In these settings, information regarding the risk of false positive results was included in pre-test counselling.

#### (iii) Repeat testing on new clients

Finally, treatment programmes that admitted patients who had been tested elsewhere, repeated the HIV test on a new sample in order to confirm the result prior to enrolling an individual for care.

#### (iv) Re-testing patients already registered in HIV programmes

Patients registered in HIV clinics prior to introduction of the new protocols were advised of the changed procedures. They were told that very occasionally RDTs falsely identify other antibodies as HIV and, therefore, confirmatory testing is recommended for all previously diagnosed HIV positive patients. Training was given to counsellors on helping individuals deal with false positive results using common scenarios and role playing. Specific information about the difference between HIV cure and a false positive result was given in the pre-test counselling to avoid misinterpretation. Psychological support was offered as long as needed, and in some cases, free health care was continued for a limited time for those found to be HIV negative.

### Data analysis

The descriptive analysis was performed using univariate analyses. Difference in means was evaluated using the Student's t-test.

### Ethical considerations

This study met the standards set by the MSF Ethics Review Board for retrospective analysis of routinely collected programmatic data. As this represents routinely collected programmatic data without personal identifiers, no individual patient consent was obtained for this retrospective review.

## Results

### Setting

#### Democratic Republic of Congo

Bukavu is a city located in eastern DRC, where MSF started an HIV programme in 2000. The initial focus was on prevention in high risk groups, and in 2002 voluntary counselling and testing (VCT) was introduced together with HIV care. Anti-retroviral treatment (ART) was made available in 2003. Testing algorithms were changed over time. Serial testing initially used Determine HIV-1/2 (Abbott Laboratories) and Capillus HIV-1/HIV-2 (Trinity Biotech) in parallel. By the end of 2004 this had evolved to Determine HIV-1/2 and Uni-Gold HIV (Trinity Biotech) in parallel [12]. Counsellors tested venous samples in front of the client and if found positive or discordant (only one test positive), a venous sample was sent to the laboratory for repeat RDTs. HIV prevalence in the testing centre was 19.3 percent (915 of 4738).

#### Burundi

Kinyinya is a rural setting in western Burundi where MSF started a small HIV programme in 2005. Testing was done in the laboratory on venous blood using a serial algorithm with Determine HIV-1/2 and Genie II HIV-1/HIV-2 (Bio-Rad) RDTs [12]. The test model was a combination of VCT and provider initiated counselling and testing (PiTC). HIV prevalence was five percent (123/2462).

#### Ethiopia

Abdurafi is a small farming community in north-western Ethiopia, where MSF supports an HIV and kala azar programme targeting migrant workers. VCT and PiCT were performed using Determine HIV -1/2 and Uni-Gold HIV in parallel on venous blood. In 2007, 2187 individuals were tested for HIV with 304 (13.9%) positive.

### Identification of individuals in HIV programmes for re-testing

#### DRC

The first programme to re-test was in DRC where the introduction of CD4 monitoring in 2004 led to a group of six patients being identified as possibly having false positive HIV diagnoses on the basis of their relatively high CD4 counts and high CD4/CD8 ratios. All six were re-tested with two RDTs and found to be negative. A further group of 54 suspect patients was identified for re-testing using screening criteria of CD4 >500 and no disease progression over one year.

#### Burundi

Confirmation testing was introduced in 2007, and retrospective testing of all previously identified positives was done as the size of the cohort was small. Prior to re-testing, the counsellors received training from an experienced counsellor on how to support individuals receiving news of being falsely diagnosed HIV positive.

#### Ethiopia

It was judged not feasible to re-test all the HIV patients in the programme at the time confirmation testing was introduced, so a high risk group was identified. Criteria were CD4 >400, clinically stable or part of a discordant couple.

### Re-testing results

#### DRC

In Bukavu, negative status was determined by repeat double negative RDTs, often with the addition of ELISA. 38 out of a total of 54 suspected false positives were found to be negative, with the remaining cases either lost to follow-up or without sufficient information on their charts to determine the final outcome.

#### Burundi

80 of 123 individuals registered in the HIV clinic were traced and 78 agreed to re-test on RDTs with the OIC test. Two (2.6%) patients were HIV negative on re-testing.

#### Ethiopia

191 patients were identified and 149 (78.0%) re-tested. Seven (4.7%) were found to be false positive on the basis of RDTs, OIC and PCR.

### Characteristics of misclassified individuals

#### DRC

Individuals identified were originally tested between 2002 and 2004. Median age was 43 years (IQR 27–46) and the male to female ratio was 0.52. Out of a total of 38 falsely diagnosed individuals, 51 percent were stage 1, 10 percent stage 2, and 38 percent stage 3 at time of entry to the programme. Amongst the 29 individuals with a recorded CD4 count, the median value was 1107 (IQR 834–1404). One result was less than 500 with a value of 473. All had CD4:CD8 ratios over 1.0.

#### Burundi

The two individuals were initially tested in 2005 and 2006. Their ages were 52 and 26 years. One was tested when she presented for medical care after sexual violence, the other tested due to TB. Both were evaluated to be clinical stage 3. CD4 counts were not available in the programme.

#### Ethiopia

All seven individuals received their original test results in 2007–8. Median age was 40 years (IQR 29.5–49.0). One was female. Amongst the seven misclassified individuals, reasons for original HIV testing were TB (one), kala azar (two), TB and kala azar (one), fever of unknown origin (two), and desire to know status (one). Six were in WHO clinical stage 3 or 4 and one in stage 1. CD4 was recorded for four individuals; three had values <500 (165, 350 and 490 respectively).

### Consequences of misclassification

#### DRC

The interval between initial positive result and being identified as negative was an average of 477 (range 17–771) days for the 38 individuals falsely diagnosed. Two women and their infants took ARV prophylaxis as part of the PMTCT programme; none started ART.

#### Burundi

The interval between the initial false positive result and being identified as negative was 577 and 828 days respectively. Both individuals started ART. One on the basis of stage 3 clinical criteria (>10% weight loss), and the other on the basis of pulmonary TB. Duration of ART was three and 18 months respectively. One individual experienced severe ART side-effects leading to a change in medications.

#### Ethiopia

The interval between initial positive result and being identified as negative was an average of 460 (range 87–610) days for the seven individuals falsely diagnosed. Four of the seven were on ART at the time of the negative diagnosis with a mean duration of 12.5 months (range 2–19 months).

### Reaction to learning of their misclassification

#### DRC

The immediate reaction to the news that they had been misdiagnosed as HIV positive was recorded by the counsellor in 21 of 38 cases (55.3%). 17 (80.9%) reactions were classified as happy, three as unhappy, and one woman was not surprised as she was already aware of her status. Amongst this group, the relief they felt was tempered for some by concern that they would no longer receive free health care. For others, being suddenly diagnosed as HIV negative affected their relationship. One woman married to an HIV positive man had been having unprotected sex for three years. A leader in the peer support network had been divorced by her HIV negative husband and re-married another HIV positive man on being diagnosed HIV positive. Others were concerned that everyone knew their status as HIV positive. Amongst the three individuals who were initially unhappy to receive the news, one woman was upset as she had stopped breastfeeding and in addition, was now going to be excluded from the HIV support group. Another was suspicious that the results were being used to exclude her from the clinic. She was offered further counselling, and on follow up, had accepted the news. One woman's reaction was mixed, as she felt overwhelmed at the disruption caused to her life by the diagnosis and could not process the fact that she had been falsely diagnosed. The individual, who was not surprised by the results, had distrusted her initial result and had re-tested at three different testing sites. All results were negative.

#### Burundi

Both patients were described as accepting the news well. One of the individuals however expressed regret as his wife had left him when she thought he was HIV positive.

#### Ethiopia

Immediate reactions to the test results were not recorded systematically in Ethiopia. However all clients accepted their results, though several had difficulty understanding how this could have happened. Two individuals requested and received a letter from MSF to help prove to others that they had been falsely diagnosed.

### Investigation of reasons for misclassification

#### DRC

Testing was done by trained nurse counsellors who received weekly supervision from the laboratory supervisor. However, it was identified that standard operating procedures (SOPs) for performing the RDTs were not in use in the VCT, and that at times the workload and stress on the counsellors was high. Quality control was done on a monthly basis in 2002–2004; however, results for the period in question were not available to the investigators. Early in the programme there was a rupture of one of the two RDTs. ELISA tests were substituted but documentation of results was lacking. It could not be ruled out that some individuals may have been diagnosed with a single test. 13 of the 38 identified were tested during this period.

#### Burundi

A review of testing practices did not reveal any concern. High discordant rates were noted for 2006, with 280 out of 1674 (16.7%) discordant using the serial algorithm of Determine HIV-1/2 and Genie II HIV-1/HIV-2. Data was not available for 2005.

#### Ethiopia

The investigation in Ethiopia did not reveal any concerns with testing procedures during the 14 months in 2007–8 when the false diagnoses took place. Data is available for 12 months of this period. 2337 tests were done, with 277 positive (11.9%) and 118 discordant (5.0%). There was 100 percent agreement for repeat testing on a randomly selected sample of 20 tests per month. The lab identified an error in VCT results in eight out of 395 (2.0%) cases. These errors were immediately corrected so did not result in patients being misclassified.

### Impact on testing volumes

#### DRC

The identification of misclassified individuals in the programme created immediate concern with the programme's team about the public credibility of the testing programme. A press release describing the problem and the new quality control measures was released in February 2005. The monthly average number tested in the four months prior to the press release was similar to that tested in the four months immediately following the announcement (373 compared to 439 respectively, p = 0.35).

#### Burundi

Testing volumes for the four month period immediately before and after introduction of re-testing, were 432 and 703 respectively.

#### Ethiopia

Mean monthly testing volumes for the four month period immediately before and after the introduction of re-testing were similar (243 and 230 respectively, p = 0.80).

### Results of Confirmation testing

#### DRC

The false positive rate for the first five months of confirmation testing using OIC for all double positive RDTs using Determine HIV-1/2 and Uni-Gold HIV was 10.3 percent (34 of 330; 95% CI: 7.2%–14.1%) out of a total of 2864 tested.

#### Burundi

The proportion of double false positive RDTs on prospective testing using a confirmatory algorithm was zero of 43 (0%, 95% CI 0%–8.2%) tested.

#### Ethiopia

In 2008, confirmation testing was introduced and in the first 15 months 29 of 407 (7.1%, 95% CI 5.0%–10.1%) false positives were prospectively identified.

## Discussion

This analysis reveals that all three projects audited had individuals enrolled in the HIV clinic who did not have HIV. This suggests that HIV misdiagnosis is an issue likely affecting a broad range of MSF programmes, but also has relevance for programmes managed with less resources than those available to MSF-supported programmes where the risks may be augmented. We therefore urge all HIV testing programmes in these settings to seriously consider this issue when designing and monitoring their testing programmes.

It is hard to overestimate the impact on individuals and their families of being diagnosed with HIV. Consequences are many ranging from the emotional distress that accompanies the diagnosis of a potentially fatal illness to the potential negative reactions of family, community members and employers. We encountered examples of individuals being divorced or thrown out of their home on hearing of their (mis) diagnosis. Some pregnant women stopped breast-feeding, putting the infants at an increased mortality risk in these settings [13]. Individuals in our cohorts were needlessly exposed to medications associated with toxicity in the short-term, but also with unknown long-term effects in non-HIV infected people. Eligibility criteria for ART in resource-limited settings include many clinical conditions and CD4 levels that are not specific to HIV [14]. The recent trend toward earlier initiation of ART both for treatment and to prevent transmission of HIV [15] will increase the risk of initiating ART in patients who do not have HIV. Many clinicians are not aware that low CD4 counts can occur in conditions other than HIV. There are two main scenarios for this. One is in the case of acute severe illness; Feeney and colleagues report CD4 levels in 102 patients admitted to the intensive care unit, only three of whom were HIV positive. 41 percent had CD4 levels below 400, and 29 percent were below 300. The CD4/CD8 ratio, thought to be a marker of HIV infection, was less than one in 16 percent [16]. Secondly, individuals have been identified with low levels of CD4 without HIV infection, both associated with opportunistic infections and also when asymptomatic [17]–[19].

The average time spent with a false diagnosis for the 47 individuals described here was 484 days, with a range up to 1287 days. On a programmatic level, the erroneous inclusion of these individuals represents significant wasted resources. While a cost effectiveness analysis is outside the scope of this paper, these results suggest that the decision to exclude a confirmation test in the testing algorithm may be a false economy.

The reason why individuals were falsely classified as HIV positive is multi-factorial. MSF testing programmes devote significant resources to supervision and quality control. Investigation in two of the three programmes did not reveal concerns in this area. In DRC, where some issues were identified, the problem persisted after resolving the quality issues. The testing algorithms did not include the higher risk tie-breaker, and used RDTs commonly in use in the country. The high percentage of false positives identified on the MSF confirmatory algorithm in Ethiopia and DRC suggests poor performance of the RDT diagnostic algorithm as the main cause of the false positives. In Burundi, no false positives were identified in prospective testing however the numbers were small and the high discordancy rate suggests that cross reactivity was occurring. The variability in false positive rates shown in our three programmes may also result from our experience that rates of cross-reactivity vary between programmes and over time within programmes (data not shown).

The risk of false positives due to poor specificity of the screening test is higher in low prevalence areas. None of the three testing centres profiled here can be considered as low prevalence and yet problems with poor positive predictive values are illustrated in the results of the confirmatory algorithm for two centres. While there is no doubt that higher prevalence contexts will have less risk of false positives, these examples illustrate that the risk is present even in higher prevalence settings.

A postulated reason for false positives is that individuals may have falsely presented to programmes as HIV positive for secondary gain such as free health care or access to nutritional support. While this may be a cause of false positives in some programmes, it did not play a factor in these three programmes as standard practice was to re-test all those tested outside of MSF centres.

Confirmation testing was introduced to all MSF testing centres without significant disruption to services and with minimal cost. Similarly it proved possible to re-test patients enrolled in HIV clinics without negatively impacting on the testing uptake. A reason postulated to not re-test individuals in HIV programmes to confirm their diagnosis, is that it would undermine faith in the testing programme. This reasoning prioritizes the potential risk to the majority who are correctly identified as HIV positive over the harm done to the small minority falsely diagnosed. MSF's experience is that this trade-off is not necessary. In fact, in many communities, the problems with testing were well known as individuals often re-test on their own or visit other centres to ‘confirm’ their diagnosis. Communication that a new more accurate algorithm is available has actually improved people's faith in the testing programme in our experience and consequently uptake of testing has not been adversely affected, as illustrated in the three case studies. Another concern expressed was that of legal repercussions, however, MSF has experienced none despite having offered re-testing in more than 10 programmes.

This analysis has a number of limitations. The programmes used to illustrate the intervention were not chosen randomly and therefore may not accurately reflect the range of programmes. Efforts were made to choose programmes representative of MSF's projects at the time, and to include a small and a large programme, as well as an integrated programme (Burundi) and a vertical project (Ethiopia, DRC). The data were collected as part of routine programmatic activities and therefore the consistency of collection across programmes cannot be assured. There are also areas of missing data in some programmes.

## Conclusions

The false diagnosis of HIV is an under-recognized but important risk in HIV programmes in resource-limited settings with significant impacts on affected individuals. Causes are multifactorial but applicable to all programmes in these settings. We were able to improve our testing and counselling services to reduce the risk to patients and feasibly introduce repeat and confirmation testing into established programmes without undermining confidence in the testing. Considering the importance of the HIV diagnosis to the individual, as well as the resources needed to care for someone with HIV, programmes should consider investigating current cohorts to determine if some individuals are misclassified. In addition, urgent attention is needed to improving quality control procedures at testing centres, and to further pilot confirmatory algorithms that are low cost and feasible to implement in resource limited settings.
